# Chicago Medical College

**Published:** 1876-01

**Authors:** N. S. Davis


### CHICAGO COLLEGE.

Clinic by Prof. N. S. Davis, Oct. 16, 1875.

### EPILEPSY.

A boy between seven and eight years of age; robust in appearance; face a little flushed; countenance rather dull in expression, but otherwise looking well, was first presented. The mother of the boy gave his history, as follows: A little more than two years ago, on a warm summer day, he came in from play excessively heated, took a hearty meal, and lay down as if going to sleep. In a few minutes she discovered him in a severe general convulsion.

The spasms ceased in a few moments, followed by fifteen or twenty minutes of apparent sleep, when he awoke and soon appeared to be well. He had no return of similar paroxysms during the next eight or ten months. After this they began to recur without any apparent cause, and have returned more and more frequently, until at present he has from one to six or seven attacks each day. Each paroxysm commences with sudden loss of consciousness, twitching of the muscles of the face and eyes, and general muscular rigidity, arresting the respiration until the face becomes purple or livid; then relaxation commences, accompanied by irregular, noisy respiration, sometimes forcing frothy saliva from the mouth, but in a few moments the breathing becomes natural, the purplish hue leaves the face, and after ten or fifteen minutes of quiet, he stands up as if waking from sleep, and appears quite well. He is often attacked in the midst of his play, and after recovering his consciousness, returning to it as if nothing had happened, and without any recollection of what had transpired during the paroxysm. The mother says his bowels move generally once or twice a day, and she thinks the urine natural in quantity, though sometimes scanty. His appetite for hearty food is voracious, and he is well nourished. She does not remember that he had any fits or other severe sickness in infancy.

When he begins to recover consciousness after his paroxysms, he rubs his nose and face, which has led to the idea that he had worms, but none have been discovered in his evacuations.

The Professor remarked that this was a good representative of the most common form of epilepsy, as it occurs in young subjects.

But all cases of epilepsy are not alike, either in their origin, progress or results. Some originate in irritation established in some part of the periphery of the sentient nerves, while others are caused by primary irritation in the brain. The first have been called *excentric*, and the latter centric or *concentric*.

Mechanical injury to the extremity of some sentient nerve, or its inclusion in the contraction of a cicatrix from previous injuries ; irritation of the alimentary canal from worms or indigestible food, and morbid conditions of the sexual organs, are the more frequent causes of excentric cases of epilepsy. Cases arising from the first mentioned cause may occur at any period of life ; those from irritation of the alimentary canal are limited chiefly to childhood ; while the last mentioned are most apt to be manifested near the period of puberty, and in females the paroxysms generally occur at the beginning or just after the close of the menstrual periods. In cases of excentric epilepsy, the mental faculties do not become impaired as readily, or to so great a degree, as in cases dependent on primary morbid action in the brain; and the leading object of the treatment is to remove the cause involving the peripheral portion of the sentient nerves. When such cause is capable of removal the prognosis is favorable. Those cases of epilepsy, however, which originate from a primary morbid condition of some portion of the brain, though most apt to begin in childhood, may originate at any period of life; and, when once begun, the paroxysms generally increase in frequency the longer they are permitted to continue. In this variety of cases the mental faculties generally become impaired in direct ratio to the frequency of the paroxysms and the duration of the disease. When the disease commences in infancy, or early childhood, and continues until adult age, it not only reduces the mind to a condition bordering on dementia, but it so retards the nutrition of the anterior portion of the brain as to make the front part of the head both low and narrow. The case before us was probably one of centric epilepsy, and unless it can be arrested, will probably induce all the consequences, physical and mental, to which allusion has been made. The essential nature of the disease is involved in doubt.

That there is a morbid condition of the common motor centre in the base of the brain, either primary or reflex, there can be no doubt. It is probable that such morbid condition consists in a disturbance or increase of the excitability at first, and is therefore functional. But a continuance of such exaltation of the excitability, coupled with the congestion induced by the paroxysms, leads to slow but certain changes in the molecular movements concerned in the nutrition of the part. Hence, long continuance of the disease, generally develops both permanent alterations of structure and impairments of function. In the case before us, as in all similar ones, the indications for treatment are, to allay the morbid excitability of the nervous centre to such an extent as to prevent the recurrence of the spasmodic paroxysms, and to re-establish healthy nutrition.

Until a recent period, a long list of remedies were empirically used for the cure of epilepsy. The more important of these were nitrate of silver, sulphate of zinc, sulphate of copper, arsenical preparations, and belladonna.

But since the efficacy of the bromides, as sedatives to purely nervous excitability, has become generally known to the profession, they have well nigh superseded all other remedies in the treatment of this disease. It is perhaps true that they may have come to occupy the attention of the profession too exclusively, and to be used too indiscriminately in these cases. In such cases as the one now before the class, exhibiting fullness and richness of blood, voracious appetite and active digestion, it was claimed that digitalis, ergot, and calabar bean, might be useful as adjuvants to the bromides in overcoming the morbid excitability.

The lecturer also called attention to the great advantage of properly regulating the diet of such patients.

He advised the entire omission of meat, as well as tea, coffee, and all alcoholic drinks, allowing the patient only the free use of milk, farinaceous articles, vegetables and fruit. A fair amount of habitual out-door exercise, freedom from over-excitement and mental anxiety, and thorough ventilation of the sleeping room, were also mentioned as matters of importance. Not only were all these things represented as important in the treatment of the disease, but that they must be faithfully pursued for many months, to secure permanent curative results. The following prescription was ordered for the patient, to be given in doses of one teaspoonful each morning, noon, tea-time and bed-time: ℞ Potassii Bromid., ʒ vi; Tinct. Digitalis, ʒ iv; Syrup. Prun. Virgin., ℥ jss; Aq. fluv., ℥ iv. M.

If the paroxysms become very much less frequent, the number of doses were to be reduced to three per day. The mother was enjoined to regulate the diet, exercise, etc., in strict accordance with the foregoing recommendations, and to bring the patient back in two weeks for further instruction.

